# Application of Funnel Metadynamics to the Platelet Integrin αIIbβ3 in Complex with an RGD Peptide

**DOI:** 10.3390/ijms25126580

**Published:** 2024-06-14

**Authors:** Robert E. Coffman, Tamara C. Bidone

**Affiliations:** 1 Scientific Computing and Imaging Institute, University of Utah, Salt Lake City, UT 84112, USA; rcoffman@sci.utah.edu; 2 Department of Biomedical Engineering, University of Utah, Salt Lake City, UT 84112, USA; 3 Department of Biochemistry, University of Utah, Salt Lake City, UT 84112, USA; 4 Department of Molecular Pharmaceutics, University of Utah, Salt Lake City, UT 84112, USA

**Keywords:** metadynamics, integrin, molecular modeling, enhanced sampling simulations

## Abstract

Integrin α_IIb_β_3_ mediates platelet aggregation by binding the Arginyl-Glycyl-Aspartic acid (RGD) sequence of fibrinogen. RGD binding occurs at a site topographically proximal to the α_IIb_ and β_3_ subunits, promoting the conformational activation of the receptor from bent to extended states. While several experimental approaches have characterized RGD binding to α_IIb_β_3_ integrin, applying computational methods has been significantly more challenging due to limited sampling and the need for a priori information regarding the interactions between the RGD peptide and integrin. In this study, we employed all-atom simulations using funnel metadynamics (FM) to evaluate the interactions of an RGD peptide with the α_IIb_ and β_3_ subunits of integrin. FM incorporates an external history-dependent potential on selected degrees of freedom while applying a funnel-shaped restraint potential to limit RGD exploration of the unbound state. Furthermore, it does not require a priori information about the interactions, enhancing the sampling at a low computational cost. Our FM simulations reveal significant molecular changes in the β_3_ subunit of integrin upon RGD binding and provide a free-energy landscape with a low-energy binding mode surrounded by higher-energy prebinding states. The strong agreement between previous experimental and computational data and our results highlights the reliability of FM as a method for studying dynamic interactions of complex systems such as integrin.

## 1. Introduction

**Figure 1:**
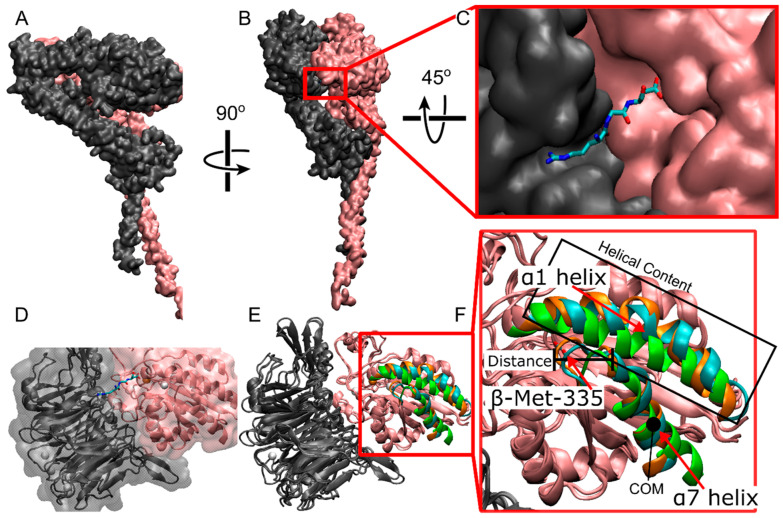
All-atom representation of α_IIb_β_3_ integrin. (**A**,**B**) Different viewpoints of full-length integrin in quick surf representation. The α_IIb_ subunit is in gray; the β_3_ subunit is in pink. (**C**) The ligand binding site at the interface between α_IIb_ β-propeller and β_3_ β-I domains (quick surf representation) with a bound RGD peptide (licorice representation). (**D**) The ligand binding site with the representation in C is made transparent and overlaid with integrin (new cartoon representation). (**E**) Overlay of the closed cryo-EM structure (Cyan) used in this study with the reference closed (Orange, PDBID: 3t3p [15]) and open (Green, PDBID: 2VDR [16]) structures. Specific atoms and regions are highlighted within the β-I domain of the β_3_ chain. These structures are known to show the biggest changes between closed and open crystal structures. (**F**) zoomed in view of the β-I domain in E. Gray and pink represent the α_IIb_ and β_3_ subunits, respectively. In the ligand (**C**,**D**), cyan = carbon, blue = nitrogen, red-oxygen. (**D**,**E**) white spheres = Ca^2+^, orange sphere = Mg^2+^.

Integrins are transmembrane heterodimers that play a crucial role in mediating adhesions and signaling pathways between cells and their surrounding environment or other cells. Comprising α and β subunits (Figure 1A,B), there are currently 18 α and 8 β subunits known, forming a total of 24 different combinations [1]. Among these, the α_IIb_β_3_ heterodimer is expressed by blood platelets and is essential for hemostasis, platelet aggregation, and blood clot retraction, making it a significant target for antithrombotic therapies. One of the best-established paradigms in α_IIb_β_3_ integrin biology is the recognition of ligands through the Arg-Gly-Asp (RGD) sequence (Figure 1C,D). The affinity of α_IIb_β_3_ for the RGD sequence increases as conformational activation progresses from bent-closed to extended-open states [2,3]. Additionally, α_IIb_β_3_ recognizes an AGDV sequence at the C-terminus of the fibrinogen γ subunit [4], and once fibrinogen is converted to fibrin by thrombin, integrin binds other sites composed of RGD sequences [5,6,7]. RGD mimetic drugs compete with fibrinogen binding to α_IIb_β_3_. Currently, three RGD-based drugs, namely tirofiban, abciximab [8], and eptifibatide [9], are clinically used to inhibit platelet aggregation and prevent thrombosis. Their crystal structures complexed with α_IIb_β_3_ have been determined [10]. However, these drugs act as partial agonists [11,12], leading to increased thrombosis in some patients and exposure to neo-epitopes, causing an immune reaction that results in thrombocytopenia in others [13]. RGD ligand mimetics that stabilize a water molecule within the β_3_ subunit create a stable closed conformation of the binding site, inhibiting large-scale conformational changes [14]. Assessing the structural rearrangements of the integrin binding site during RGD binding and evaluating the formation and disassembly of molecular contacts in a dynamic setting will provide insights for the future design of RGD-based drugs to inhibit conformational activation and prevent thrombosis.

The binding of an RGD peptide to α_IIb_β_3_ integrin is a complex dynamic process involving structural changes of the ligand binding site (Figure 1E,F), accompanied by RGD motions and several sequential interactions of RGD with different integrin residues [17]. RGD binding initiates molecular rearrangements of the α_IIb_ β-propeller and β_3_ β-I domains forming the ligand binding site (Figure 1E,F). These rearrangements include a lateral movement of the backbone of β-Ser-123 in the β1-α1 loop, a downward shift of the α7 helix, a downward lateral motion of the carbonyl oxygen of β-Met-335 in the β6-α7 loop, and an increase in helicity in the α1 helix [17]. These rearrangements change the position of the β-propeller domain, resulting in the opening of the headpiece and an increase in affinity for ligand binding [18,19,20,21]. Unfortunately, experimental methods that can capture these rearrangements at high spatial and temporal resolutions are lacking, and computational modeling approaches present limitations in dynamic sampling. 

Experiments primarily based on crosslinking studies, loop swapping, site-directed mutagenesis, and soaked crystals using fibrinogen and ligand mimetic monoclonal antibodies have identified several residues directly involved in establishing the molecular interactions at the α_IIb_ β-propeller and β_3_ β-I domains with an RGD peptide, but not their dynamics. Crystal structure and cyrogenic electron microscopy (cryo-EM) studies using a variety of peptide ligands have identified critical contacts, including α-Asp-159, α-Phe-160, α-Tyr-189, α-Tyr-190, α-Leu-192, α-Asp-224, α-Ser-225, α-Phe-231, α-Asp-232, β-Ser-121, β-Tyr-122, β-Ser-123, β-Ser-213, β-Arg-214, β-Asn-215, and β-Arg-216, (α = α_IIb_, β = β_3_ subunits [15,16,17,22,23,24,25]. Other integrin residues contact the RGD peptide exclusively through a mediating water molecule; these include α-Asp-163, β-Tyr-166, β-Ala-218, and β-Asp-251 [15,16,17,22,25]. Alanine scanning mutation and binding studies identified three additional contacts in both integrin subunits: α-Arg-165, α-Tyr-166, and α-Phe-191 [26,27]. 

Studies based on molecular modeling have captured some aspects of the dynamics of RGD binding and unbinding to α_IIb_β_3_ integrin or the closely related α_V_β_3_ integrin. However, these approaches presented limitations due to high-energy barriers that separate different binding modes, making transitions between them rare events. These methods have utilized steered molecular dynamics (SMD), quantum calculations, molecular docking, umbrella sampling (US), and equilibrium molecular dynamics (MD) simulations [28,29,30,31,32,33,34,35,36,37]. The strongest RGD interaction energies with the α_IIb_ and β_3_ subunits were identified as follows: the Mg^2+^ metal ion-dependent adhesion site (MIDAS) ion, Ca^2+^ adjacent to MIDAS (ADMIDAS), and ligand-associated metal binding site (LIMBS) ions, α-Asp-224, α-Asp-159, β-Asn-215, and β-Lys-125 [30]. MD studies have reported a few integrin contacts that were not shown through crystal structure or cryo-EM studies, including β-Ala-252, α-Ser-161, and β-Asp-217 [29,32,35,37]. Insights into the RGD binding pathway energy, the formation of hydrogen bonds, and optimal distances between Asp-410 and the MIDAS Mg^2+^ ion were also provided [36]. However, none of these modeling approaches utilized more than one biased variable to enhance sampling and cross high energy barriers. They also imposed positional restraints on either the integrin or the RGD peptide, or both [38,39,40,41] and did not simulate sequential binding and unbinding events that occur in physiological conditions [17]. 

In this study, we employed all-atom simulations using funnel metadynamics (FM) to enhance the sampling sufficiently to evaluate the dynamic interactions of an RGD peptide with the α_IIb_ β-propeller and β_3_ β-I domains of integrin. FM does not require a priori information about the interactions between RGD and integrin and incorporates an external history-dependent potential while applying a funnel-shaped restraint potential to limit RGD exploration of the unbound state. FM also provides structural flexibility for both protein and ligand and includes explicit water molecules for solvation and desolvation. It also considers conformational selection and induced-fit effects, which often play a role in identifying ligand binding modes [42]. Our FM simulations showed that RGD binding to α_IIb_β_3_ integrin triggers structural rearrangements within the β_3_ β-I domain. Furthermore, our simulations provided a free-energy landscape with a low-energy binding mode surrounded by higher-energy prebinding states, demonstrating the existence of multiple binding modes. Our analysis also revealed several critical interaction residues distributed across the α_IIb_ and β_3_ subunits, aligning closely with findings from previous studies. In sum, this study demonstrates the effectiveness of funnel metadynamics in precisely evaluating the dynamics of RGD binding to α_IIb_β_3_ integrin.

## 2. Results

### 2.1. Structural Comparison between Cryo-EM and Reference Crystal Structures of Integrin α_IIb_β_3_

The objective of this analysis was to evaluate the structural properties of the β-propeller and β-I domains of cryo-EM integrin α_IIb_β_3_ used in this study by comparing these domains with the closed conformer (PDBID: 3T3P [15]) as a reference. The β-I domain of cryo-EM α_IIb_β_3_ was structurally aligned to the β-I domain of the reference crystal, resulting in a Cα RMSD of 0.071 nm. The distances between specific backbone atoms in the cryo-EM α_IIb_β_3_ and the corresponding atoms in the reference structure were measured: β-Met-335 oxygen (0.707 Å), α7 helix center of mass (COM) (0.955 Å), and β-Ser-123 nitrogen (0.915 Å). These comparisons confirm the cryo-EM structure’s comparability to the reference crystal. 

### 2.2. Structural Dynamics of Integrin β-I Domain: Insights from Funnel Metadynamics Simulations

Given that RGD binding to α_IIb_β_3_ triggers molecular rearrangements in the β-I domain that propagate to the α subunit and to the lower legs for conformational activation [10,43,44], our objective was to evaluate the efficacy of funnel metadynamics (FM) simulations in capturing the mechanisms. Ten independent walkers were employed for FM, with those reaching 150 ns considered for structural analysis. Additionally, three 150 ns equilibrium molecular dynamics (MD), along with a 1 µs equilibrium MD simulation without RGD and divalent cations were run for comparison. 

First, we examined the evolution of the helical content in the α1 helix. FM walkers with RGD and divalent cations displayed an increase in average helical content from ~70% to ~77% (Figure S1A,B). Equilibrium MD simulations on the same system showed variable results, with two out of three simulations exhibiting a decrease in helical content to ~53% and one showing an increase to ~76% (Figure S1C), suggesting that the presence of RGD and divalent cations can promote integrin activation but with lower probability than with biased ligand binding. Conversely, without divalent cations and RGD, the equilibrium simulation showed a decrease in average helical content to 63% (Figure S1D).

Next, we analyzed the dynamics of the α7 helix and the backbone oxygen atom of β-Met-335 in the β-I domain. We compared their distances from the corresponding positions in closed (PDB ID: 3T3P [15]) and open (PDB ID: 2VDR [16]) crystal structures. During FM simulations, these distances decreased, indicating a shift of the α7 helix and β-Met-335 from the bent towards the open state of integrin (Figures S2A,B and S3A,B). Equilibrium simulations on the same systems displayed more stable measures (Figures S2C and S3C), while the equilibrium simulation without divalent cations and RGD showed motion towards the closed crystal reference instead of the open (Figures S2D and S3D).

Overall, our analysis revealed that RGD binding and divalent cations are crucial for increasing helical rearrangements of the α1 helix and repositioning of the α7 helix and β-Met-335 away from the ligand binding interface of α_IIb_β_3_ integrin in its bent closed conformation, towards their positions in the extended-open state. These findings affirm the capability of FM to capture molecular rearrangements of the β-I domain induced by RGD binding and the influence of divalent cations on these dynamics.

### 2.3. Assessing Sampling Efficiency: Evaluation of Funnel Metadynamics Simulations

**Figure 2:**
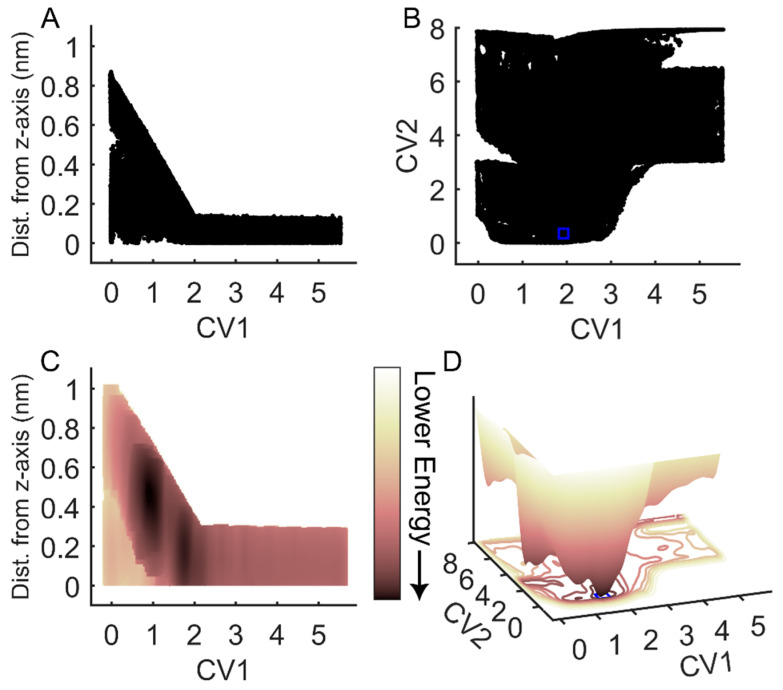
Exploration of the RGD peptide of the funnel and biased CV space during FM simulations. (**A**) RGD center of mass (COM) exploration within the funnel space. (**B**) RGD exploration in the space of the biased collective variables (CVs), with CV1 representing RGD COM along the funnel *z*-axis and CV2 indicating the difference in contact maps. (**C**) Two-dimensional projection of the reweighted free energy surface of RGD COM within the funnel space. (**D**) Three-dimensional representation of RGD energy as a function of the biased collective variables in (**C**). The blue squares in panels (**B**,**D**) denote the minimum of the free energy surface, which is used to determine CV space boundaries for frame extraction and contact analysis. The arrow indicates the direction of decreasing energy: lighter colors correspond to higher energy values; darker colors correspond to lower energy values.

To assess the efficacy of FM for simulations of the binding and unbinding between an RGD peptide and α_IIb_β_3_ integrin, we conducted an evaluation of sampling efficiency across two key dimensions: the funnel space and the biased collective variable (CV) space (CV1 and CV2). Examination of the cumulative exploration of the ligand binding site for the RGD peptide, defined by the projections of the center of mass (COM) and contacts of RGD across all FM walkers, revealed a comprehensive exploration of both the funnel space and the biased CV space (Figure 2A,B). In the solvated state, where the RGD peptide exhibited no contact with the ligand binding site, the free energy surface appeared flat (refer to Figure 2C,D for CV1 = 3–5 nm). Conversely, in the bound state, a distinct energy well emerged, with the minimum corresponding to CV1 = 1.5 nm and CV2 = 0.1–0.3 (Figure 2D). Through reweighted free energy analysis within the funnel space, we determined that the positions of the free energy minima were sufficiently distant from the funnel edges, suggesting that the funnel did not overly restrict the ligand within the binding site (Figure 2C). Overall, our findings demonstrate that FM offers robust sampling efficiency, ensuring a comprehensive exploration of the conformational landscapes across several free energy barriers.

### 2.4. Evaluation of RGD Interactions with the Binding Site of α_IIb_β_3_ Integrin

**Figure 3:**
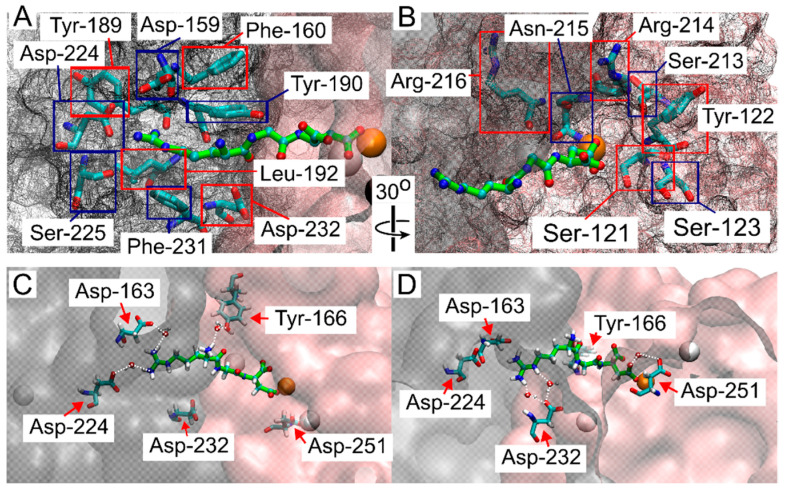
Structural analysis of the interactions between the RGD peptide and the ligand binding site of α_IIb_β_3_ integrin. (**A**) Residues in the α_IIb_ domain and (**B**) in the β-I domain that interact with the RGD peptide, as identified in this study and consistent with crystal structure and cryo-EM studies [15,16,27,29,30,32,33,34]. Red and blue squares indicate the labeled integrin residues, while the β-propeller and β-I domains are represented by a gray mesh and a pink mesh, respectively. The RGD peptide is shown in green, with atoms shown as small spheres (**C**,**D**). Contacts reported to be water-mediated in previous structural studies [16,17,22,25] and confirmed here, with β-propeller and β-I domains represented in gray and pink, respectively, with a transparent surface. The RGD peptide is shown in green, with atoms shown as small spheres. The color scheme is as follows: oxygen in red, carbon in cyan, nitrogen in blue, hydrogen (on residues) or Ca^2+^ (as a large sphere) in white, and Mg^2+^ represented by an orange sphere.

Because the RGD peptide explored the integrin binding site within the funnel’s cone, as well as the solvated state further from the cone, and did not experience bias from the funnel while in the bound state (Figure 2), we aimed to determine where binding occurred. Frames from FM simulations were extracted from all walkers where the COM of the RGD ligand was in close contact with the binding site around 1.5 nm on CV1, and close to 0 distance from reference contacts (CV2), and within 10 kJ/mol of the lowest energy point, (this region is indicated by a blue rectangle in Figure 2B,D). These extracted frames were used for all successive analyses. A contact between RGD and integrin was considered formed when a heavy atom within the RGD peptide and a heavy atom within a residue of the β-propeller or β-I domains were within 5 Å of each other [45]. Many of the contacts reported by crystal structures and cryo-EM studies were confirmed in this study (Figure 3A,B). These contacts, in order of highest to lowest percentage of frames exhibiting the contacts, were: α-Tyr-190 (100%), MIDAS Mg^2^⁺ (100%), α-Phe-160 (99%), α-Tyr-189 (99%), β-Ser-121 (99%), β-Arg-214 (98%), β-Asn-215 (76%), α-Phe-231 (71%), α-Asp-159 (69%), β-Ser-213 (66%), β-Arg-216 (58%), α-Asp-224 (58%), β-Tyr-122 (44%), α-Leu-192 (43%), α-Ser-225 (31%), β-Ser-123 (31%), and α-Asp-232 (6%).

It has been previously reported that RGD contact with residues α-Asp-224 and α-Asp-232 is occasionally mediated by a water molecule, depending on the specific ligand and the conformation of integrin [16,17,22,25]. To estimate the role of water in the integrin α-Asp-224 and α-Asp-232 contacts, the number of extracted contact frames where a single water molecule was detected within a specified distance (4, 3.3, and 2.6 Å) for both contacting residues was counted (Supplemental Table S1). For the α-Asp-224 and Arg-408 contact, the percentage of frames with water within 4, 3.3, and 2.6 Å were 55%, 40%, and 0.12%, respectively. For the α-Asp-232 and Arg-408 contact, the percentages were 100%, 99%, and 0.5%, respectively. Contacts reported to be exclusively mediated by water in crystal structure or cryo-EM studies but exhibiting direct contact within our criteria were β-Ala-218 (97%), β-Asp-251 (56%), α-Asp-163 (31%), and β-Tyr-166 (14%) (Figure 3C,D) [16,17,22,25]. These contacts were also evaluated for water H-bond estimates (Supplemental Table S1). β-Ala-218 showed the most contact with Asp-410, with 84%, 43%, and 0% of the contact frames containing a water molecule within the respective distance criteria. β-Asp-251 showed 99%, 91%, and 5% of contact frames with a water molecule within the respective distances, and α-Asp-163 showed 100%, 98%, and 0.2% of contact frames with a water molecule within the respective distances. β-Tyr-166 showed the most contact with ligand residue Arg-408, with 99.5%, 88%, and 10% of contact frames showing a water molecule within the respective distances. β-Tyr-166 and β-Ala-218 showed contact with the other two ligand residues but to a much lesser extent (Supplemental Table S1). 

Other contacts not identified in the crystal structure or cryo-EM studies but described in MD studies [29,32,35,37] and confirmed in this study were: β-Ala-252 (72%), α-Ser-161 (71%), β-Asp-217 (51%), α-Ser-226 (8%), and α-Trp-162 (6%). Additionally, ligand contact with α-Arg-165, α-Tyr-166, and α-Phe-191, reported in alanine scanning and ligand binding studies, were found in 0.51%, 6%, and 1.4% of the extracted frames, respectively (Table 1 and Figure S4). Collectively, results from our simulations demonstrate that FM simulations of RGD binding and unbinding of α_IIb_β_3_ integrin accurately capture binding modes for this complex system, validating the effectiveness of FM for simulations of complex protein/ligand systems.

## 3. Discussion

The interplay between the α_IIb_ and β_3_ subunits in establishing the molecular contacts of integrin with an RGD peptide has been thoroughly explored using a variety of experimental and computational techniques, including X-ray crystallography, cryo-EM, MD simulations, crosslinking studies, loop swapping, and site-directed mutagenesis. While experimental methods provide invaluable insights, computational approaches offer higher spatial and temporal resolution, making them crucial for capturing detailed ligand-protein interactions, especially in a dynamic setting. Previous computational studies, however, have encountered limitations such as restricted sampling and the need for a priori information on integrin/RGD binding modes. These approaches have struggled to accurately capture the full transitions between unbound and bound states of the ligand, limiting their ability to identify complex binding modes. This study demonstrates the utility of funnel metadynamics in capturing structural changes at the binding site and exploring complex binding modes in a dynamic setting. 

Application of FM to the integrin/RGD complex resulted in topological rearrangements within the β_3_ subunit β-I domain (Figure S1), indicating that the transitions of the ligand between a solvated and a ligated state underlie integrin conformational activation. Consistent with this result, previous reports have shown that structural changes in the β-I domain correlate with the conformational transitions of integrin from inactive to active states [3,18,20]. In particular, as ligand binding occurs and integrin moves from a bent-closed to an extended-open conformation, the hybrid domain swings out, and within the β-I domain, the helicity of the α1 helix increases, the oxygen atom of β-Met-335 moves away from the binding site, and the α7 helix drops down [16,17,25,48], all supportive of our results. 

Prior computational investigations relied on methodologies such as steered molecular dynamics (SMD), umbrella sampling (US), quantum mechanics (QM), and equilibrium molecular dynamics (MD). SMD simulations have been employed to extract the ligand from its binding site, facilitating the identification of contact-breaking events and the determination of the RGD unbinding force [28,29,31,32,36]. Additionally, SMD has been utilized to drive an RGD peptide into the binding site, enabling the estimation of binding pathway energy, hydrogen bond formation, and the final distance between specific residues such as Asp-410 and the MIDAS Mg^2+^ ion [36]. Among the interactions investigated, those involving ligand residues Arg-408 and Asp-410, and α-Asp-224 and the MIDAS Mg^2+^ ion, respectively, have been consistently identified as pivotal binding interactions within the α_IIb_β_3_ integrin binding site. However, it is noteworthy that SMD simulations have typically employed pulling forces larger than physiological values. US simulations, on the other hand, have constrained the ligand’s center of mass at various distances from the protein, allowing exploration of other degrees of freedom. Nonetheless, these simulations have not adequately distinguished the free energy differences attributable to these degrees of freedom. Equilibrium MD simulations have often initiated the ligand within the binding site; however, due to limited sampling, unbinding events have not been adequately captured [31,33,34]. Conversely, starting the RGD ligand outside of the binding site has facilitated the observation of binding events but not unbinding [32]. Quantum mechanical (QM) calculations have identified residues with the highest RGD interaction energies from the α_IIb_ and β_3_ chains, including residues such as α-Asp-224, α-Asp-159, β-Asn-215, and β-Lys-125, as well as the MIDAS Mg^2+^ ion, and the Ca^2+^ ions in the ADMIDAS and LIMBS sites [30]. However, the inflexibility of the protein within the docking program, coupled with the computational complexity of QM calculations, has limited their ability to account for dynamic rearrangements of the target protein [38,39,40,41]. None of these methods have fully captured the complete transitions of RGD between unbound and bound states, which are crucial for identifying complex binding modes. 

Several residues are known to interact with an RGD peptide from the α_IIb_ and β_3_ subunits [15,16,27,29,30,32,33,34]. Our FM simulations identified numerous residues involved in these interactions, including α-Tyr-190, α-Phe-160, α-Tyr-189, β-Ser-121, β-Arg-214, β-Asn-215, α-Phe-231, α-Asp-159, β-Ser-213, β-Arg-216, β-Tyr-122, α-Leu-192, β-Ser-123, and others (Table 1 and Figure 3A,B). This corroborates the reliability of FM as a tool for studying the integrin/RGD complex. Notably, FM captured the contact between the RGD residue Asp-410 and the MIDAS Mg^2+^ ion in all frames, suggesting its crucial role in integrin activation. It is conceivable that the establishment of this strong contact causes an AGDV peptide, lacking the α_IIb_ binding residue, to promote headpiece opening [22]. The contact between Arg-408 and α-Asp-224 was observed less frequently (53%), often mediated by water molecules (Supplemental Table S1 and Figure 3C), as found in previous reports [16,17,22,25]. Similar mediation by water molecules was observed for the contact between Arg-408 and α-Asp-232 (Figure 3D). Water molecules increase the distance between interacting residues, complicating direct contact detection. Certain contacts reported as water-mediated in previous studies, such as those involving β-Ala-218, β-Tyr-166, β-Asp-251, and α-Asp-163 [15,16,17,22,25,29,33,37], were detected as direct contacts in this study. β-Ala-218 showed significant contact with Asp-410, Gly-409, and Arg-408, with water molecules frequently present within 4 Å of these contacts (Supplemental Table S1). β-Tyr-166, located deep in the binding site, maintained stable water-mediated contact when the contact was present (Figure 3C). β-Asp-251, and α-Asp-163 water mediated contacts were less certain. Additional contacts identified by FM include β-Ala-252, α-Ser-161, and β-Asp-217, all also described in computational studies based on MD simulations [29,32,35,37]. For instance, β-Ala-252 contacts a ligand Asp sidechain oxygen atom, forming a weak hydrogen bond [49,50]. New insights were gained into contacts involving α-Arg-165, α-Tyr-166, α-Phe-191, and α-Ser-226, as previously found [26]. These contacts were infrequent but occasionally observed, highlighting the dynamic nature of integrin-ligand interactions.

To conclude, this study demonstrates the utility of funnel metadynamics in exploring structural rearrangements of the ligand binding site and integrin-RGD peptide interactions. It reveals molecular changes in the β_3_ subunit of integrin upon RGD binding and provides a free-energy landscape with a low-energy binding mode surrounded by higher-energy prebinding states. Identifying both known and novel contacts enhances our understanding of ligand recognition mechanisms for α_IIb_β_3_ integrin. The findings corroborate previous observations while providing additional molecular details, particularly regarding water-mediated interactions and rarely observed contacts. Overall, the strong agreement between previous experimental and simulation data and our results highlights the reliability of FM as a method for studying complex protein/ligand systems. Funnel metadynamics could be applied in future research to inform therapeutic strategies targeting protein/ligand interaction processes. 

## 4. Methods

### 4.1. Structure Setup for Equilibrium, Steered MD, and Funnel Metadynamics 

The bent-closed conformation of the full-length integrin α_IIb_β_3_, derived from crystal structures and fitted into cryo-electron microscopy (cryo-EM) density maps, was used for our investigations [2]. This structure was modified by adding missing residues [3]. The amino acid sequence was changed using CHARMM-GUI PDB Reader and Manipulator [51,52,53] to match the canonical sequence at uniprot.org, α_IIb_ P08514-1 and β_3_ P05106-1 [54]. 

We isolated key components, namely the α_IIb_ β-propeller domain (residues 1 to 452), the β_3_ β-I domain (residues 109 to 351), and the RGD peptide in its bound state (residues 408–410), using Visual Molecular Dynamics (VMD) software for WIN64, version 1.9.4a53, University of Illinois at Urbana-Champaign, USA [55]. Subsequently, the introduction of divalent cations was executed using a custom VMD script, referencing PDBID 3ZDY [17] to align residues surrounding each ion’s binding site. Specifically, calcium ions were aligned with distinct groups of residues originating from the α_IIb_ β-propeller domain, forming group 1 (434, 432, 426, 428, 430), group 2 (371, 365, 367, 369, 373), group 3 (299, 297, 303, 305, 301), and group 4 (245, 250, 247, 252, 243). The remaining calcium ions (2 Ca^2+^) and magnesium ion (Mg^2+^) were incorporated into the β_3_ β-I domain, aligning with residues as follows: group 1 (219, 217, 158, 220, 215), group 2 (127, 126, 123), and for the Mg^2+^ ion, group 3 (220, 121). In previous research, these divalent cations have been identified as critical for ligand binding [56,57]. This system was used for SMD and FM simulations and for the three replicas of the equilibrium simulations with RGD present.

To prepare the structure for simulation, we utilized the CHARMM-GUI Lehigh University, Bethlehem, PA, USA, Solution Builder [51,58]. The setup included a pH of 7.0, employing the CHARMM36m force field [59], applying standard N and C-terminal group patching for all three protein chains, solvating with the CHARMM-modified TIP3P water model [60], and introducing 0.15 M Sodium Chloride (NaCl) to neutralize the charge of the system, using the Monte-Carlo placement method. The resulting system consisted of 162,986 atoms with 10,598 protein atoms, 50,696 water molecules, and 143 Cl, 150 Na, 6 Ca^2+^, and 1 Mg^2+^ ions, arranged within a 120 × 120 × 120 Å box. 

Subsequently, the system underwent minimization and equilibration using the GROMACS 2022.5 software, Science for Life Laboratory, Stockholm, SE [61,62,63]. Lennard-Jones interactions were truncated at 1.2 nm with the Verlet switching function, ranging from 1.0 to 1.2 nm. Long-range electrostatic interactions were calculated using the particle mesh Ewald (PME) summation method with a 0.12 nm grid spacing [64]. The systems were minimized using the steepest descent algorithm for 5000 steps or until a maximum force of 1000 kJ mol^−1^ nm^−1^ was reached, with a step size of 0.01 nm. Positional restraints for both minimization and equilibration were set at 400 kJ mol^−1^ nm^−2^ for protein backbone atoms and 40 kJ mol^−1^ nm^−2^ for protein side chain atoms. Subsequently, the temperature was raised to 310 K using velocity rescaling with the Nose–Hoover thermostat and equilibrated for 125,000 steps with 0.001 ps timesteps (125 ps total), implementing the canonical ensemble (NVT), which maintains a constant number of atoms, volume, and temperature. Subsequently, positional restraints were released, and production runs were conducted in the isothermal-isobaric ensemble (NPT), which maintains a constant pressure of 1 atm using isotropic Parrinello–Rahman coupling [65] and a temperature of 310 K using the Nose–Hoover thermostat. Covalent bonds involving hydrogen atoms were constrained with the LINCS algorithm [66], allowing for a timestep of 0.002 ps. This equilibrated system was used to produce three replicas of equilibrium simulations of 150 ns each. Additionally, to facilitate steered molecular dynamics (SMD) and subsequent funnel metadynamics (FM) simulations, a restraint based on a harmonic potential with a spring constant of 10 kJ/mol was applied to the alpha carbon (Cα) atoms in residues 96 and 107 within the β-propeller domain. These residues were chosen based on the evaluation of root mean squared fluctuation (RMSF) of individual integrin Cα atoms over the first 50 ns of equilibrium MD simulations, which indicated that these atoms exhibit the lowest fluctuation across the three replicas.

### 4.2. Steered Molecular Dynamics Simulations

The MOVINGRESTRAINT bias, implemented in PLUMED 2.8.2, was utilized to extract the RGD ligand from the binding site while maintaining the funnel restraint. PLUMED is an open-source, community-developed library [67]. The resulting trajectory served as the basis for initializing the walkers. The restraint was initiated near the ligand’s initial center of mass (COM) position at 0.4 nm and gradually extended to 4.5 nm over 2,500,000 steps (5 ns) with a linear increase in the spring constant during this period. Initially set at 0 kJ/mol, the spring constant was progressively raised to 1000 kJ/mol at the 5 ns mark before reverting to 0 kJ/mol at the 10 ns mark. The total simulation time was 7 ns. Frames and PLUMED output were saved every 500 steps (1 ps). Further details on the funnel setup are elaborated in the subsequent section.

### 4.3. Funnel Metadynamics Simulations

**Figure 4:**
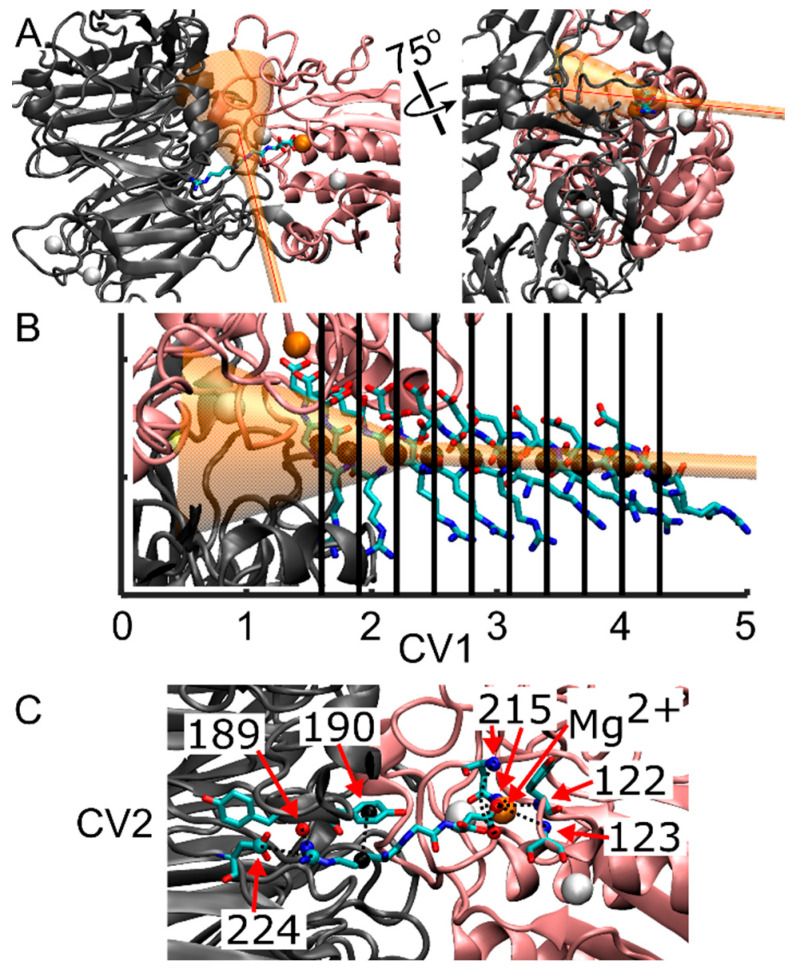
Representation of the integrin/ligand complex including the funnel structure, and indication of the collective variables for FM simulations. (**A**) New cartoon representation of the α_IIb_ β-propeller domain (gray) and the β_3_ β-I domain (pink), forming the ligand binding site. The RGD peptide is in licorice representation, while the funnel is shown in transparent orange. The first collective variable (CV1), corresponding to the funnel *z*-axis, is shown with a red line. (**B**) Initial spatial positions of the RGD peptide for the various simulation walkers along CV1. The black sphere denotes the center of mass (COM) of the RGD peptide along the funnel *z*-axis, as derived from SMD, serving as the starting coordinates for the RGD ligand for each of the ten walkers. (**C**) Contacts considered in the calculation of the second collective variable (CV2). These contacts are formed between residues from the α_IIb_ subunit that interact with Arg-408 (α-Tyr-189, α-Tyr-190, α-Asp-224), residues from the β_3_ subunit that interact with Asp-410 (β-Asn-215, β-Tyr-122, β-Ser-123), and the Mg^2+^ ion that interacts with Asp-410. Dashed lines represent the eight contacts utilized to measure the deviation, in distance, between the initial reference contacts and those observed throughout the simulation. Note that β-Asn-215 forms two interactions with Aps-410. Atoms are color-coded as follows: oxygen (red), carbon (cyan), nitrogen (blue), calcium (white spheres), magnesium (orange sphere), and center of mass of atoms forming contacts with RGD (black spheres).

Following the Steered Molecular Dynamics (SMD) protocol, a series of multiple walker well-tempered Funnel Metadynamics (FM) simulations were conducted using GROMACS 2022.5, integrated with PLUMED 2 [68]. This approach employs multiple walkers with a “cone and cylinder” or funnel-shaped restraint, in conjunction with well-tempered metadynamics, aimed at enhancing the sampling within the target ligand binding site and its solvated state. The funnel constraint was imposed solely when the ligand approached the edge of the funnel, as depicted in Figure 4A,B. Simultaneously, the cylinder allowed controlled exploration of the solvated state, thereby increasing the sampling of RGD in both bound and solvated states. To prevent the RGD ligand from diffusing into the bulk solvent, a restraint in the form of a harmonic upper wall was positioned at the end of the cylinder, capped at 5.5 nm from the funnel anchor with a kappa value of 35,100. All parameters governing the funnel restraint were manually selected utilizing the Funnel-Metadynamics Advanced Protocol (FMAP-GUI) plugin [42] in VMD 1.9.4.a53. 

The anchoring point and origin of the funnel were defined as atom number 7655, corresponding to a Cα atom on β-Pro-163 in the β-I domain, thereby centering the cone on the α_IIb_β_3_ binding site. The second funnel point was chosen on the docked ligand to ensure alignment of the α_IIb_β_3_ binding site and bound ligand within the funnel. Specific funnel parameters were set as follows: a cylinder radius of 1.0 Å, an amplitude of 0.35 radians for the cone section, and a switching point between the cone and the cylinder set at 2.0 nm. The center of mass (COM) calculation of the ligand’s position along the *z*-axis of the funnel utilized all heavy atoms within the RGD ligand chain (residue 408 to 410).

Multiple walker metadynamics allowed concurrent contributions from multiple simulations (walkers) to the metadynamics bias, thereby enhancing sampling efficiency within a shorter real-time duration. Ten walkers were initiated from points within the SMD trajectory, each commencing at a frame nearest to the beginning of the SMD simulation and spaced at regular 0.3 nm intervals along the funnel, as illustrated in Figure 4B, ranging from 1.6 nm to 4.1 nm. Walker 5, initiated at 3.1 nm, attained a simulation time of 121 ns, revealing an error in calculating the funnel grid, potentially stemming from inadequate restraints confining the ligand within the funnel boundaries or adjustments in the funnel leading to ligand placement outside the funnel grid between timesteps. Each of these walkers, except where noted above, reached 150 ns.

Two collective variables (CVs) were selected to drive the metadynamics bias: CV1, representing the center of mass (COM) position of the RGD peptide along the funnel’s *z*-axis, and CV2, measuring the distance from a reference contact map (unitless) (Figure 4B,C). These contacts involved residues from the α_IIb_ subunit that interact with Arg-408 (α-Tyr-189, α-Tyr-190, α-Asp-224), residues from the β_3_ subunit that interact with Asp-410 (β-Asn-215, β-Tyr-122, β-Ser-123), and the Mg^2+^ ion that interacts with Asp-410 (Figure 4C). 

CV2 called the CONTACTMAP within PLUMED, was computed using a rational switching function, defined as:

$$s\left(r\right)=\frac{1-{\left(\frac{r\;-\;d_{0}}{r_{0}}\right)}^{n}}{{1-(\frac{r\;-\;d_{0}}{r_{0}})}^{m}}$$

where d_0_ = 0.0, n = 6, m = 2n, r_0_ = 1 nm is the switch distance, and r is the distance of the atom from its contact pair. Each switched distance is then squared and summed, and the resulting value is then subtracted from the same calculation with the switched reference values. This equation, known as the CMDIST function within PLUMED, is written as:

$${(s}_{ref1}^{2}+s_{ref2}^{2}+s_{ref3}^{2}+\cdots )-{(s}_{1}^{2}+s_{2}^{2}+s_{3}^{2}+\cdots )$$

where S_ref1,ref2,ref3,…_ are the switched output of the reference contacts and S_1,2,3…_ are the switched output of the same contacts from the current timestep. The first CV allows the bias to further enhance sampling of the solvated and bound state, and the second CV was chosen to distinguish different binding modes of the ligand in the binding site, if present. 

The Gaussian deposition rate was every 500 steps (1 ps) with a height of 2.0 kJ/mol and a width of 0.1 nm for CV1 and 0.5 for CV2, based on equilibrium simulations. The bias factor was set to 20. Additionally, the WHOLEMOLECULES feature was employed in PLUMED 2 for all protein atoms to facilitate the reconstruction of these molecules within PLUMED in case of crossing the periodic boundary, thereby preventing calculation artifacts. Output from PLUMED and coordinates (frames) from GROMACS were saved every 500 steps (1 ps). 

### 4.4. Equilibrium Molecular Dynamics Simulations without Divalent Cations and RGD

The full-length α_IIb_β_3_ integrin structure from [2], modified to match the canonical sequence at uniprot.org, was embedded into a membrane and solvated at pH 7.0 using CHARMM-GUI Membrane Builder [51]. To align the protein with the membrane plane, the Orientation Option “Run PPM 2.0” was employed, taking input from both integrin subunits. A lipid ratio of 7:3 POPE:POPC (palmitoyl-oleoyl-phosphatidylethanolamine:palmitoyl-oleoyl-phosphatidylcholine) was utilized, resulting in a membrane configuration similar to cellular membranes. Sodium chloride at a concentration of 0.15 M was incorporated via the distance ion placing method. The resulting system comprised 359,339 atoms, with 540 lipid molecules, 87,641 water molecules, 292 Na ions, and 233 Cl ions, arranged within a box of dimensions 132 × 132 × 217 Å. Note that no divalent cations or RGD ligands were included.

The system underwent minimization using the steepest descent algorithm for 5000 steps or until a maximum force of 1000 kJ mol^−1^ nm^−1^ was reached, with a step size of 0.01 nm, while maintaining four restraints on the protein and lipids equal to those applied during six equilibration steps. Specifically, the position restraint on the lipid phosphorus atom followed a series of 1000, 400, 400, 200, 40, and 0 kJ/mol. Several dihedral angles in the lipids were restrained in a series of 1000, 400, 200, 200, 100, and 0 kJ/mol. The protein backbone atoms were restrained in a series of 4000, 2000, 1000, 500, 200, and 50 kJ/mol, while the protein sidechain atoms were restrained in a series of 2000, 1000, 500, 200, 40, and 0 kJ/mol. These equilibration steps were conducted over different durations and timestep sizes: the first three steps endured for 125 ps at 1 fs timesteps, while the final three steps were each extended by 500 ps at 2 fs timesteps. The Berendsen thermostat maintained the temperature at 310 Kelvin during equilibration, switching to the Nose–Hoover thermostat for production runs. All restraints were released for production runs, with consistent application of cutoff scheme, PME calculations, NPT ensemble, and LINCS algorithm, mirroring parameters utilized in the funnel metadynamics simulations. This system underwent simulation for 1 µs with a timestep of 2 fs.

MDANALYSIS [69,70] and MDTRAJ [71,72] were used for trajectory analysis of both equilibrium and enhanced simulations. Details are provided in the Supplementary Information.

## Figures and Tables

**Table 1:** Comparison between α_IIb_β_3_ integrin residues interacting with RGD in this study and previous investigations. The first column provides the list of residues from the two integrin subunits. The second column lists the percentage of frames capturing the contact. This percentage is calculated from the FM free energy surface as the total number of frames in which the RGD was found in the bound state. The third column provides references to previous studies that have demonstrated the existence of the contacts. * indicates residues with contacts for RGD that are water-mediated.

Integrin Residues Interacting with RGD	Frames (%)	References
**α_IIb_ chain**		
Glu-157	0	[34]
Asp-159	69%	[29,30,34]
Phe-160	99%	[15,17,22,29]
Ser-161	71%	[29]
Trp-162	6%	[29]
Asp-163 *	31%	[17,22,29]
Arg-165	0.51%	[27]
Tyr-166	6%	[27]
Tyr-189	99%	[16,17,23,24,27,37]
Tyr-190	100%	[15,16,17,29,33,37]
Phe-191	1.4%	[27]
Leu-192	43%	[15,16]
Asp-224	53%	[15,16,17,22,25,30,33,34,37]
Ser-225	31%	[17,23,25]
Ser-226	8%	[27,37]
Glu-229	0	[34,37]
Phe-230 (Tyr-230)	0 (0.001%)	[37]
Phe-231	71%	[15,16,17,22,29]
Asp-232 *	6%	[15,16,17,22,23,25,32,33,34,37]
Tyr-234	0	[37]
Trp-260	0	[37]
Ser-161	0	[37]
Arg-281	0	[34]
**β_3_ chain**		
Asp-119	100%	Binds Mg^2+^
Leu-120	46%	false positive
Ser-121	99%	[22,23,29,35]
Tyr-122	44%	[16,17,22,31,35,36]
Ser-123	31%	[16,17,22,23,29,35,36]
Lys-125	0	[30,36]
Lys-126	0	[36]
Asp-127	0	[29,32,34]
Val-157	8.4%	false positive
Tyr-164	0	[46]
Tyr-166 *	14%	[17,33]
Glu-171	0	[32]
Ser-211	0	[32]
Ser-213	66%	[22,47]
Arg-214	98%	[16,17,29,30,31,36,37]
Asn-215	76%	[15,16,17,22,29,30,31,35,36]
Arg-216	58%	[17,22,23,25,36]
Asp-217	51%	[35,37]
Ala-218 *	97%	[15,16,17,22,25,29,33,37]
Glu-220	100%	[15,33]
Gly-222	0	[32]
Thr-250	41%	false positive
Asp-251 *	56%	[29,33]
Ala-252	72%	[29]
Glu-312	0	[32,34]
Asn-313	0	[29,32]
Mg^2+^ ion (MIDAS)	100%	[16,17,22,23,30,31,34,36]

## Data Availability

Simulation inputs, trajectories and analysis scripts are available at https://github.com/tamarabidone/funnel_metadynamics_aIIBb3_RGD.

## Supplementary Materials

The following supporting information can be downloaded at https://www.mdpi.com/article/10.3390/ijms25126580/s1.

## References

[1] Hynes R.O. (2002). Integrins: Bidirectional, allosteric signaling machines. Cell.

[2] Xu X.-P., Kim E., Swift M., Smith J.W., Volkmann N., Hanein D. (2016). Three-dimensional structures of full-length, membrane-embedded human αiibβ3 integrin complexes. Biophys. J..

[3] Tong D., Soley N., Kolasangiani R., Schwartz M.A., Bidone T.C. (2023). Integrin αiibβ3 intermediates: From molecular dynamics to adhesion assembly. Biophys. J..

[4] Yang Z., Kollman J.M., Pandi L., Doolittle R.F. (2001). Crystal structure of native chicken fibrinogen at 2.7 Å resolution. Biochemistry.

[5] Zafar H., Shang Y., Li J., David G.A., Fernandez J.P., Molina H., Filizola M., Coller B.S. (2017). A_iib_β_3_ binding to a fibrinogen fragment lacking the γ-chain dodecapeptide is activation dependent and edta inducible. Blood Adv..

[6] Litvinov R.I., Farrell D.H., Weisel J.W., Bennett J.S. (2016). The platelet integrin αiibβ3 differentially interacts with fibrin versus fibrinogen. J. Biol. Chem..

[7] Sánchez-Cortés J., Mrksich M. (2009). The platelet integrin αiibβ3 binds to the RGD and AGD motifs in fibrinogen. Chem. Biol..

[8] Coller B.S. (1997). Platelet gpiib/iiia antagonists: The first anti-integrin receptor therapeutics. J. Clin. Investig..

[9] Musial J., Niewiarowski S., Rucinski B., Stewart G.J., Cook J.J., Williams J.A., Edmunds L.H. (1990). Inhibition of platelet adhesion to surfaces of extracorporeal circuits by disintegrins. RGD-containing peptides from viper venoms. Circulation.

[10] Xiao T., Takagi J., Coller B.S., Wang J.-H., Springer T.A. (2004). Structural basis for allostery in integrins and binding to fibrinogen-mimetic therapeutics. Nature.

[11] Pullarkat R.K. (1991). Hypothesis: Prenatal ethanol-induced birth defects and retinoic acid. Alcohol. Clin. Exp. Res..

[12] Hantgan R.R., Stahle M.C. (2009). Integrin priming dynamics: Mechanisms of integrin antagonist-promoted αiibβ3: PAC-1 molecular recognition. Biochemistry.

[13] Bougie D.W., Wilker P.R., Wuitschick E.D., Curtis B.R., Malik M., Levine S., Lind R.N., Pereira J., Aster R.H. (2002). Acute thrombocytopenia after treatment with tirofiban or eptifibatide is associated with antibodies specific for ligand-occupied gpiib/iiia. Blood.

[14] Lin F.-Y., Li J., Xie Y., Zhu J., Huong Nguyen T.T., Zhang Y., Zhu J., Springer T.A. (2022). A general chemical principle for creating closure-stabilizing integrin inhibitors. Cell.

[15] Zhu J., Choi W.-S., McCoy J.G., Negri A., Zhu J., Naini S., Li J., Shen M., Huang W., Bougie D. (2012). Structure-guided design of a high-affinity platelet integrin aiibb3 receptor antagonist that disrupts MG2+ binding to the midas. Sci. Transl. Med..

[16] Springer T.A., Zhu J., Xiao T. (2008). Structural basis for distinctive recognition of fibrinogen γc peptide by the platelet integrin αiibβ3. J. Cell Biol..

[17] Zhu J., Zhu J., Springer T.A. (2013). Complete integrin headpiece opening in eight steps. J. Cell Biol..

[18] Tvaroška I., Kozmon S., Kóňa J. (2023). Molecular modeling insights into the structure and behavior of integrins: A review. Cells.

[19] Slack R.J., Macdonald S.J.F., Roper J.A., Jenkins R.G., Hatley R.J.D. (2022). Emerging therapeutic opportunities for integrin inhibitors. Nat. Rev. Drug Discov..

[20] Kolasangiani R., Bidone T.C., Schwartz M.A. (2022). Integrin conformational dynamics and mechanotransduction. Cells.

[21] Huang J., Li X., Shi X., Zhu M., Wang J., Huang S., Huang X., Wang H., Li L., Deng H. (2019). Platelet integrin αiibβ3: Signal transduction, regulation, and its therapeutic targeting. J. Hematol. Oncol..

[22] Lin F.-Y., Zhu J., Eng E.T., Hudson N.E., Springer T.A. (2016). Β-subunit binding is sufficient for ligands to open the integrin αiibβ3 headpiece. J. Biol. Chem..

[23] Adair B.D., Xiong J.-P., Yeager M., Arnaout M.A. (2023). Cryo-em structures of full-length integrin αiibβ3 in native lipids. Nat. Commun..

[24] Zhu J., Luo B.-H., Xiao T., Zhang C., Nishida N., Springer T.A. (2008). Structure of a complete integrin ectodomain in a physiologic resting state and activation and deactivation by applied forces. Mol. Cell.

[25] Zhu J., Zhu J., Negri A., Provasi D., Filizola M., Coller B.S., Springer T.A. (2010). Closed headpiece of integrin αiibβ3 and its complex with an αiibβ3-specific antagonist that does not induce opening. Blood.

[26] Kamata T., Irie A., Tokuhira M., Takada Y. (1996). Critical residues of integrin αiib subunit for binding of αiibβ3 (glycoprotein iib-iiia) to fibrinogen and ligand-mimetic antibodies (pac-1, op-g2, and lj-cp3)*. J. Biol. Chem..

[27] Kamata T., Tieu K.K., Irie A., Springer T.A., Takada Y. (2001). Amino acid residues in the αiib subunit that are critical for ligand binding to integrin αiibβ3 are clustered in the beta-propeller model. J. Biol. Chem..

[28] Craig D., Gao M., Schulten K., Vogel V. (2004). Structural insights into how the midas ion stabilizes integrin binding to an RGD peptide under force. Structure.

[29] Kononova O., Litvinov R.I., Blokhin D.S., Klochkov V.V., Weisel J.W., Bennett J.S., Barsegov V. (2017). Mechanistic basis for the binding of RGD- and AGDV-peptides to the platelet integrin αiibβ3. Biochemistry.

[30] Kóňa J. (2023). Comparative study of interaction energies between αiibβ3 integrin and the peptidic, peptidomimetic and non-peptidic ligands by quantum mechanics FMO-PIEDA calculations. Chem. Pap..

[31] Murcia M., Jirouskova M., Li J., Coller B.S., Filizola M. (2008). Functional and computational studies of the ligand-associated metal binding site of β_3_ integrins. Proteins Struct. Funct. Bioinform..

[32] Mehrbod M., Trisno S., Mofrad Mohammad R.K. (2013). On the activation of integrin αiibβ3: Outside-in and inside-out pathways. Biophys. J..

[33] Li J., Vootukuri S., Shang Y., Negri A., Jiang J.-k., Nedelman M., Diacovo T.G., Filizola M., Thomas C.J., Coller B.S. (2014). RUC-4 a novel αiibβ3 antagonist for prehospital therapy of myocardial infarction. Atertio. Thromb. Vasc. Biol..

[34] Zhao L., Zhai J., Zhang X., Gao X., Fang X., Li J. (2016). Computational design of peptide-Au cluster probe for sensitive detection of α_iib_ β_3_ integrin. Nanoscale.

[35] Ghitti M., Spitaleri A., Valentinis B., Mari S., Asperti C., Traversari C., Rizzardi G.P., Musco G. (2012). Molecular dynamics reveal that isoDGR-containing cyclopeptides are true αvβ3 antagonists unable to promote integrin allostery and activation. Angew. Chem. Int. Ed..

[36] Li N., Qiu S., Fang Y., Wu J., Li Q. (2021). Comparison of linear vs. Cyclic RGD pentapeptide interactions with integrin αvβ3 by molecular dynamics simulations. Biology.

[37] Arzani H., Rafii-Tabar H., Ramezani F. (2022). The investigation into the effect of the length of RGD peptides and temperature on the interaction with the αiibβ3 integrin: A molecular dynamic study. J. Biomol. Struct. Dyn..

[38] Yang Y., Yao K., Repasky M.P., Leswing K., Abel R., Shoichet B.K., Jerome S.V. (2021). Efficient exploration of chemical space with docking and deep learning. J. Chem. Theory Comput..

[39] Friesner R.A., Murphy R.B., Repasky M.P., Frye L.L., Greenwood J.R., Halgren T.A., Sanschagrin P.C., Mainz D.T. (2006). Extra precision glide:  Docking and scoring incorporating a model of hydrophobic enclosure for protein−ligand complexes. J. Med. Chem..

[40] Halgren T.A., Murphy R.B., Friesner R.A., Beard H.S., Frye L.L., Pollard W.T., Banks J.L. (2004). Glide:  A new approach for rapid, accurate docking and scoring. 2. Enrichment factors in database screening. J. Med. Chem..

[41] Friesner R.A., Banks J.L., Murphy R.B., Halgren T.A., Klicic J.J., Mainz D.T., Repasky M.P., Knoll E.H., Shelley M., Perry J.K. (2004). Glide:  A new approach for rapid, accurate docking and scoring. 1. Method and assessment of docking accuracy. J. Med. Chem..

[42] Raniolo S., Limongelli V. (2020). Ligand binding free-energy calculations with funnel metadynamics. Nat. Protoc..

[43] Adair B.D., Yeager M. (2002). Three-dimensional model of the human platelet integrin αiibβ3 based on electron cryomicroscopy and X-ray crystallography. Proc. Natl. Acad. Sci. USA.

[44] Huo T., Wu H., Moussa Z., Sen M., Dalton V., Wang Z. (2024). Full-length αiibβ3 cryo-em structure reveals intact integrin initiate-activation intrinsic architecture. Structure.

[45] Abdel-Azeim S., Chermak E., Vangone A., Oliva R., Cavallo L. (2014). Mdcons: Intermolecular contact maps as a tool to analyze the interface of protein complexes from molecular dynamics trajectories. BMC Bioinform..

[46] D’Souza S.E., Ginsberg M.H., Burke T.A., Lam S.C.-T., Plow E.F. (1988). Localization of an ARG-GLY-ASP recognition site within an integrin adhesion receptor. Science.

[47] Charo I.F., Nannizzi L., Phillips D.R., Hsu M.A., Scarborough R.M. (1991). Inhibition of fibrinogen binding to GP iib-iiia by a GP iiia peptide. J. Biol. Chem..

[48] Cheng M., Li J., Negri A., Coller B.S. (2013). Swing-out of the β_3_ hybrid domain is required for α_iib_β_3_ priming and normal cytoskeletal reorganization, but not adhesion to immobilized fibrinogen. PLoS ONE.

[49] Sutor D.J. (1963). 204. Evidence for the existence of C–H⋯O hydrogen bonds in crystals. J. Chem. Soc. (Resumed).

[50] Desiraju G.R. (1996). The C−H···O hydrogen bond:  Structural implications and supramolecular design. Acc. Chem. Res..

[51] Jo S., Kim T., Iyer V.G., Im W. (2008). CHARMM-GUI: A web-based graphical user interface for CHARMM. J. Comput. Chem..

[52] Jo S., Cheng X., Islam S.M., Huang L., Rui H., Zhu A., Lee H.S., Qi Y., Han W., Vanommeslaeghe K., Karabencheva-Christova T. (2014). Chapter eight—CHARMM-GUI PDB manipulator for advanced modeling and simulations of proteins containing nonstandard residues. Advances in Protein Chemistry and Structural Biology.

[53] Park S.-J., Kern N., Brown T., Lee J., Im W. (2023). CHARMM-GUI PDB manipulator: Various pdb structural modifications for biomolecular modeling and simulation. J. Mol. Biol..

[54] Consortium T.U. (2022). Uniprot: The universal protein knowledgebase in 2023. Nucleic Acids Res..

[55] Humphrey W., Dalke A., Schulten K. (1996). VMD: Visual molecular dynamics. J. Mol. Graphics.

[56] Raborn J., Fu T., Wu X., Xiu Z., Li G., Luo B.-H. (2013). Variation in one residue associated with the metal ion-dependent adhesion site regulates αiibβ3 integrin ligand binding affinity. PLoS ONE.

[57] Xia W., Springer T.A. (2014). Metal ion and ligand binding of integrin α5β1. Proc. Natl. Acad. Sci. USA.

[58] Lee J., Cheng X., Swails J.M., Yeom M.S., Eastman P.K., Lemkul J.A., Wei S., Buckner J., Jeong J.C., Qi Y. (2016). CHARMM-GUI input generator for NAMD, GROMACS, AMBER, OpenMM, and CHARMM/OpenMM simulations using the CHARMM36 additive force field. J. Chem. Theory Comput..

[59] Huang J., Rauscher S., Nawrocki G., Ran T., Feig M., de Groot B.L., Grubmüller H., MacKerell A.D. (2017). CHARMM36m: An improved force field for folded and intrinsically disordered proteins. Nat. Methods.

[60] Jorgensen W.L., Chandrasekhar J., Madura J.D., Impey R.W., Klein M.L. (1983). Comparison of simple potential functions for simulating liquid water. J. Chem. Phys..

[61] Bekker H., Berendsen H., Dijkstra E., Achterop S., Vondrumen R.v., Vanderspoel D., Sijbers A., Keegstra H., Renardus M. GROMACS-a parallel computer for molecular-dynamics simulations. Proceedings of the 4th International Conference on Computational Physics (PC 92).

[62] Van Der Spoel D., Lindahl E., Hess B., Groenhof G., Mark A.E., Berendsen H.J.C. (2005). GROMACS: Fast, flexible, and free. J. Comput. Chem..

[63] Abraham M.J., Murtola T., Schulz R., Páll S., Smith J.C., Hess B., Lindahl E. (2015). Gromacs: High performance molecular simulations through multi-level parallelism from laptops to supercomputers. SoftwareX.

[64] Darden T., York D., Pedersen L. (1993). Particle mesh ewald: An N⋅log(N) method for ewald sums in large systems. J. Chem. Phys..

[65] Parrinello M., Rahman A. (1981). Polymorphic transitions in single crystals: A new molecular dynamics method. J. Appl. Phys..

[66] Hess B., Bekker H., Berendsen H.J.C., Fraaije J.G.E.M. (1997). LINCS: A linear constraint solver for molecular simulations. J. Comput. Chem..

[67] Bonomi M., Bussi G., Camilloni C., Tribello G.A., Banáš P., Barducci A., Bernetti M., Bolhuis P.G., Bottaro S., Branduardi D. (2019). Promoting transparency and reproducibility in enhanced molecular simulations. Nat. Methods.

[68] Tribello G.A., Bonomi M., Branduardi D., Camilloni C., Bussi G. (2014). PLUMED 2: New feathers for an old bird. Comput. Phys. Commun..

[69] Michaud-Agrawal N., Denning E.J., Woolf T.B., Beckstein O. (2011). MDANALYSIS: A toolkit for the analysis of molecular dynamics simulations. J. Comput. Chem..

[70] Gowers R.J., Linke M., Barnoud J., Reddy T.J., Melo M.N., Seyler S.L., Domanski J., Dotson D.L., Buchoux S., Kenney I.M. MDANALYSIS: A python package for the rapid analysis of molecular dynamics simulations. Proceedings of the 15th Python in Science Conference.

[71] McGibbon Robert T., Beauchamp Kyle A., Harrigan Matthew P., Klein C., Swails Jason M., Hernández Carlos X., Schwantes Christian R., Wang L.-P., Lane Thomas J., Pande Vijay S. (2015). MDTRAJ: A modern open library for the analysis of molecular dynamics trajectories. Biophys. J..

[72] Kabsch W., Sander C. (1983). Dictionary of protein secondary structure: Pattern recognition of hydrogen-bonded and geometrical features. Biopolymers.

