# Supplementary material for: Application of Funnel Metadynamics to the Platelet Integrin αIIbβ3 in Complex with an RGD Peptide
Source: Int J Mol Sci. 2024 Jun 14;25(12):6580. doi: 10.3390/ijms25126580 (PMC11203998; doi:10.3390/ijms25126580)
Supplement: Supplementary file 1 [file ijms-25-06580-s001.zip › ijms-2956192-supplementary.pdf]

Supplementary Information

Comparison with Reference Closed and Open $\alpha_{IIb}\beta_3$ Crystals

The reference closed and open crystal structures were taken from PDB ID 3T3P [15] and 2VDR [16], respectively, and converted to a CHARMM readable format using PDB Reader and Manipulator in CHARMM-GUI for use in analysis.

Alignment for Root Mean Squared Deviation

All root mean squared deviation (RMSD) measurements used the RMSD Trajectory Tool within VMD. The C_α RMSD of the β-I domain, compared to the reference structure PDB ID 3T3P [15], was calculated after structural fit of the C_α atoms in the β-I domain. The C_α RMSD of the α7 helix was performed after aligning the C_α atoms of the α7 helix (residues 338 to 351) in the β-I domain. The C_α RMSD of the α1 helix was performed after aligning the C_α atoms within residues 127 to 146 in the β-I domain.

Distance and Secondary Structure Measurements

For distance measurements of the oxygen atom of β-Met-335 and the COM of the α7 helix, the alpha carbon atoms of residues 113 to 119, and 149 to 331 in the β-I domain were used for alignment. These residues were chosen to remove the β6-α7 loop, α7 helix and α1 helix from alignment before measurements were taken. These distances were monitored post-production with a custom VMD script.

The secondary structure of the β-I domain was monitored for all walkers of all conformations using a custom python script that used MDANALYSIS [69,70] to extract the desired residues from trajectories and MDTraj [71] for secondary structure analysis using DSSP and the simplified 3-category structure assignment scheme [72].

CV Space Monitoring

CV space graphs were created from sampling the CV space of the saved trajectories using PLUMED DRIVER. The graphs were made by creating one graph with all data from the walkers.

Binding Site Identification on the Free Energy Surface

The binding site was identified as a rectangle space on the Free Energy Surface within 10 kJ/mol of the lowest energy point near Z = 1.5 nm and Contacts = 0. The energy was Z-axis ≥ 1.4768 and ≤ 1.79464 nm and Contacts ≥ 0 and ≤ 0.52767. The solvation space is defined as Z-axis ≥ 4 and ≤ 5 nm and Contacts = 7.93563 ± the contact binsize. The

solvation space being solvated around 7.93563 of CV2 was determined by finding the lowest energy space between $4 \leq Z \leq 5$ nm.

The trajectory was reweighted using the PLUMED 2.0 REWEIGHT_BIAS algorithm with all 4 biases as input. The 4 biases are from the upper and lower walls used to keep the ligand in the funnel, metadynamics, and the funnel. The funnel free energy surface shown in Figure 2 was calculated using the HISTOGRAM function with the space of the funnel as inputs and a stride of 50. The grids were set to -1 to 6 nm for the funnel z-axis and 0 to 1.2 for the distance from the z-axis with a total of 500 bins each. The bandwidth was set to 0.05 for both variables. Once the grid was completed the CONVERT_TO_FES algorithm was used to convert the histogram to a free energy surface.

Extraction of Frames

Frames were extracted based on the lowest energy point within the measured free energy surface (Figure 3) closest to a value of 1.5 for CV1 and 0 for CV2 (Figure 3). This was considered the bound state of the RGD ligand. The extracted frames were analyzed for contacts based on a distance of 5 Å between any heavy atom within the ligand and the protein, reported previously [45]. The bound contact time was slightly altered from Abdel-Azeim, Chermak [45] and was calculated as follows:

$$contacts = (nc_{kl}/N) * 100\%$$

where contacts are percentage of bound frames, nc_{kl} is the total number of frames where residues k of protein A and l of protein B are in contact, N is the total number of analyzed frames. So, a value of 100% is interpreted as all extracted frames showing contact between the two designated residues and 0% means no contact.

The VMD snapshot renderer was used for all snapshots shown in this report. All graphs were plotted using MATLAB.

Analysis of Water Contacts

The role of water in specific contacts were assessed by determining how many frames with contact met another criterion: a water molecule was found within distance of both contacting residues and their oxygen and nitrogen atoms.

Supplemental Table 1. Contacts identified in this study, known to be mediated by water molecules, indicate the presence of water molecules between integrin and RGD residues. R = Arg, G = Gly or D = Asp below the integrin residue represents the ligand residue it contacts. Reported are the percentage of frames where the indicated contact was made and had a water molecule within the specified distance of both residues.

	**α-Asp-224**<br>**R**	**α-Asp-232**<br>**R**	**β-Ala-218**<br>**R (G) D**	**β-Asp-251**<br>**D**	**α-Asp-163**<br>**R**	**β-Tyr-166**<br>**R (G) D**
4.0 Å	100 %	100 %	55 (17) 84 %	99 %	100 %	99.5 (91) 99 %
3.3 Å	100 %	99 %	40 (6) 43 %	91 %	98 %	88 (42) 95 %
2.6 Å	0.2 %	0.5 %	0.1 (0) 0 %	5 %	0.2 %	10 (0) 2 %
Contact Frames	5340	600	844 (9906) 9058	5821	3318	1441 (541) 150

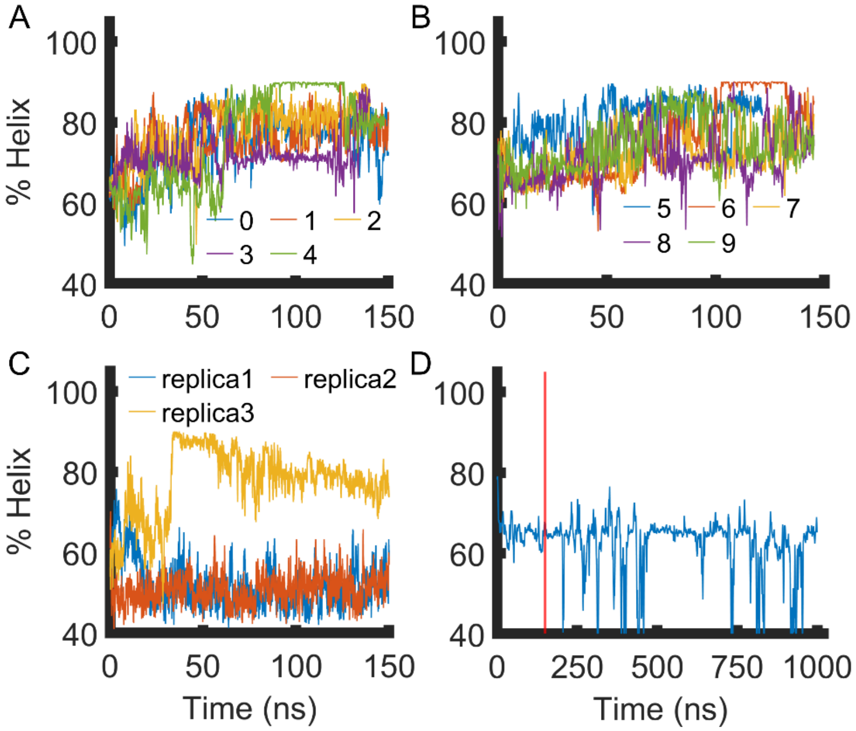

Supplemental Figure 1. The α1 helical content in the β₃ subunit β-I domain increases in FM simulations with RGD and divalent cations but decreases in equilibrium simulations without RGD. (**A**) FM Walkers 0 through 4 and (**B**) 5 through 9. (**C**) Equilibrium simulations with divalent cations and RGD ligand (replicas 1 through 3). (**D**) Equilibrium simulation without divalent cations or RGD, with the red vertical line marking 150 ns.

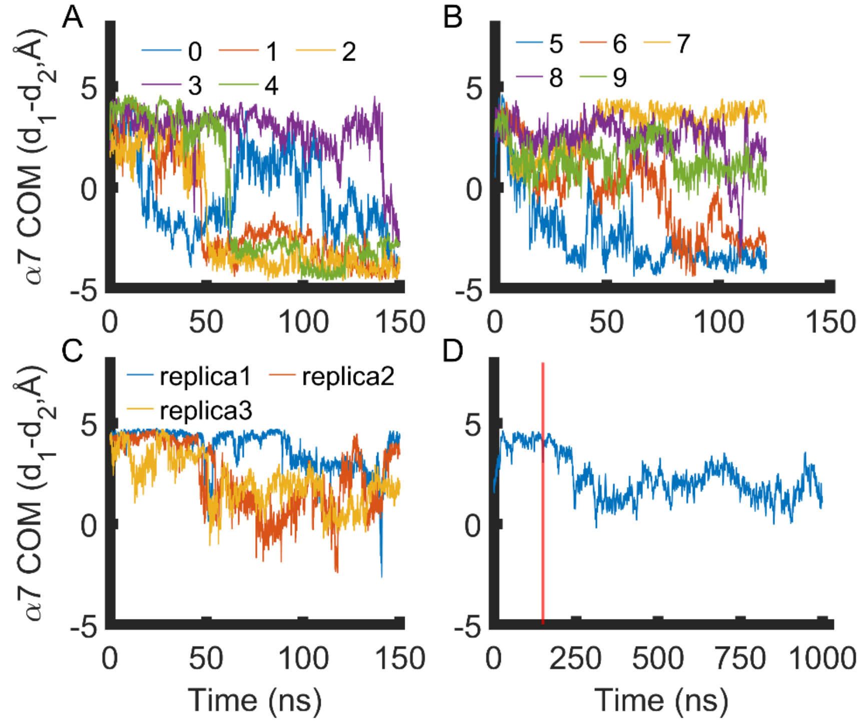

Supplemental Figure 2. The α7 helix COM in the β-I domain moves closer to the open reference structure during FM and equilibrium MD simulations. d1 and d2 are the distances of the center of mass (COM) of the α7 helix from the reference closed (PDB ID 3T3P [15]) and open (PDB ID 2VDR [16]) structures, respectively. Plotted is the difference of these distances, i.e. at 5 Å the group is closest to the closed structure, at 0 Å the group is equidistant from both structures and at -5 Å the group is closest to the open structure. (**A**) FM walkers 0 through 4 and (**B**) 5 through 9. (**C**) Equilibrium MD simulations with divalent cations and RGD ligand. (**D**) Equilibrium MD simulation with no divalent cations and no RGD ligand, with the red vertical line marking 150 ns.

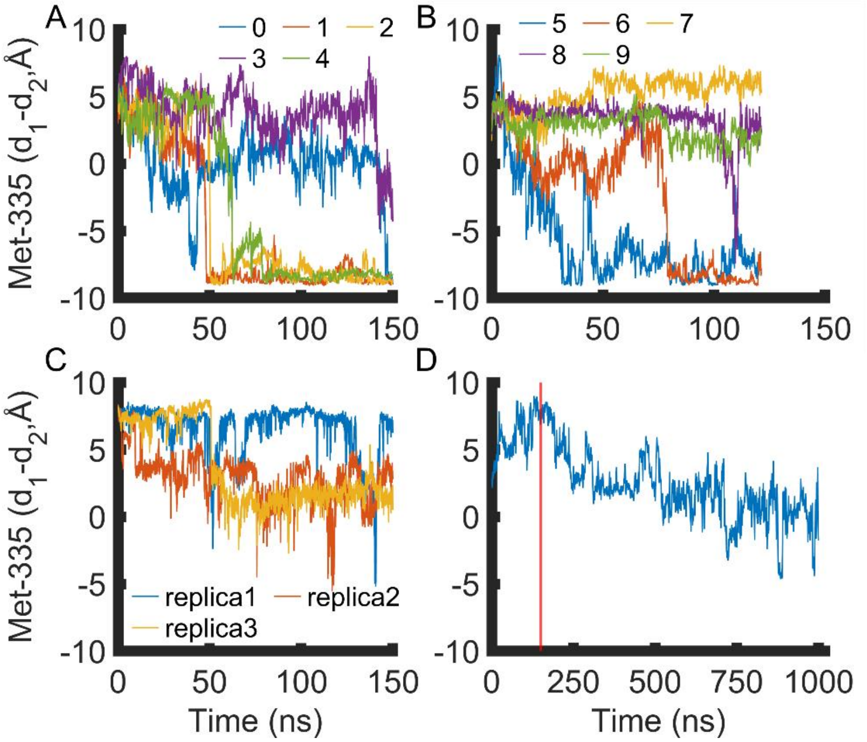

Supplemental Figure 3. The position of the Met-335 backbone oxygen atom in the β-I domain moves closer to the open reference structure during FM and equilibrium MD simulations. d1 and d2 are the distances from the reference closed and open structures, respectively. Plotted is the difference of these distances of the backbone oxygen atom of β-Met-335. I.e. at 5 Å the atom is closest to PDB ID 3T3P [15], at 0 Å the atom is equidistant from both references and at −5 Å the atom is closest to PDB ID 2VDR [16]. (**A**) FM walkers 0 through 4 and (**B**) 5 through 9. (**C**) Equilibrium MD simulation with divalent cations and RGD ligand. (**D**) Equilibrium MD simulations with no divalent cations and no RGD ligand. 150 ns highlighted as red vertical line.

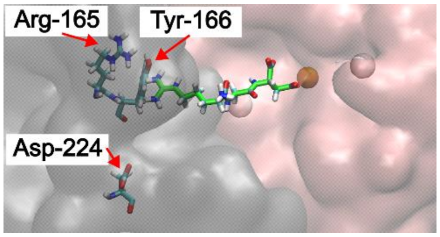

Supplemental Figure 4. Contacts found through Ala-scanning and ligand binding experiments and confirmed from this study. The ligand residue Arg-408 breaks off contact with α-Asp-224 and shifts upward into the site to contact α-Arg-165 and α-Tyr-166. Phe-191 was found to barely fall within the contact criteria of 5 Å, and not shown.
